# Impact of orthodontic treatment on temporomandibular joint function among adolescents: A longitudinal study

**DOI:** 10.6026/973206300211982

**Published:** 2025-07-31

**Authors:** Prasanth Prathapan Santhakumari, Sanu Tom Abraham, Eldho Thuruthumalil Paul, Eunice John, Alen Eldho, Vighnesh Varma Raja

**Affiliations:** 1 Department of Orthodontics & Dentofacial Orthopedics, Indira Gandhi Institute of Dental Sciences, Nellikuzhi, Kothamangalam, Ernakulam, Kerala, India; 2 Department of Orthodontics & Dentofacial Orthopaedics, Annoor Dental College and Hospital, Muvattupuzha Ernakulam, Kerala, India

**Keywords:** Orthodontic treatment, temporomandibular joint, adolescents, TMJ dysfunction, longitudinal study, RDC/TMD

## Abstract

The alteration in the functionality of the temporomandibular joints (TMJ) during adolescence among participants receiving fixed orthodontic treatment over the period of 12 months is of interest. The sixty participants between 12-17 years were measured at baseline, 6 months and 12 months according to the RDC/TMD criteria. There were brief episodes of augmented joint noises and tenderness at 6 months and these were enhanced by 12 months. The mandibular movement increased tremendously (p = 0.032) whereas there was no TMJ degeneration in the long-term among most patients. Thus, fixed orthodontic orthotic does not affect the health of TMJ among adolescents.

## Background:

Orthodontic treatment is often started during adolescence to fix malocclusion, problems with the jaw or skull and crowded teeth. Due to the high plasticity of the bones in the head and face, treatment can be done easily in this stage. At the same time, there has been a long-standing argument in the field about how orthodontic forces influence the health of the temporomandibular joint (TMJ) [1]. Because the temporomandibular joint is complex and always in motion, it is vulnerable to changes in its function during the years when orthodontic devices are used [2]. A temporomandibular disorder (TMD) affects the way the joint, masticatory muscles and related structures function and may result in pain, noises in the joint, reduced movement or imbalances during jaw movement [3]. Not all experts think that orthodontics causes or increases TMD problems. Some research argues it may in fact be useful in certain cases of TMD dysfunction [4, 5]. The inconsistency could result from differences in how the studies were designed which conditions they investigated and the way treatments were given. Many studies recognize the RDC/TMD as a standard set of guidelines to assess people with problems in their jaw joints (TMJs) which help detect changes in these symptoms [6]. When using RDC/TMD, longitudinal studies can help us understand the way TMJ problems change or improve for orthodontic patients. The lack of long-term information on the impact of fixed orthodontics on the TMJ in adolescents was the reason this study looked at TMJ function during a 12-month period. The study uses repeated clinical exams and patient reports to help improve the evidence for orthodontic care of TMJ health. Also, in adolescence, there is fast change in the body's skeleton and muscles, making the temporomandibular joint more capable but, at the same time, potentially more vulnerable to stress [7]. Some people worry that using orthodontic forces during this phase of jaw and tooth development may interrupt the regular function of the TMJ. Although there are reports of discomfort and joint sounds during orthodontic treatment, it not always clear if these have any clinical importance. We should understand that defensive or adaptive reactions are clinically acceptable, but not to be confused with the formation of dysfunction [8, 9]. There are divergent views in the literature about a link between orthodontic work and TMD. On the other hand, some studies that last over a long period suggest that orthodontic treatment may help improve symptoms of TMJ by fixing bite problems and bettering chewing ability [10]. Therefore, it is of interest to investigate how fixed orthodontic treatment affects the temporomandibular joint status in adolescents over a defined period using validated diagnostic criteria.

## Materials and Methods:

Over the course of 12 months, researchers conducted a longitudinal observational study. The research was conducted on adolescents between the ages of 12 and 17 who were going to start fixed orthodontic treatment. Before the study started, the Institutional Review Board gave ethical approval and written informed consent was collected from all the participants and their legal guardians.

## Sample selection:

A total of 60 adolescents (30 male and 30 female) who meet the criteria were enrolled in the study. For inclusion, patients needed to (1) be between 12 and 17 years old, (2) not have any systemic disorders, (3) have never had orthodontic treatment and (4) not have a history of surgery or injury to the TMJ. Criteria for exclusion were: (1) having severe temporomandibular disorders based on RDC/TMD, (2) having craniofacial disorders and (3) being on medications that change neuromuscular function.

## Orthodontic treatment and on-going care:

Everyone was given fixed appliance therapy that involved 0.022" slot MBT brackets. Standard treatment protocols were used by using archwires as specified. Patients were not fitted with additional orthopedic appliances nor had any teeth removed during the observation period so as not to mix up the main signs. Both the dentist and the patient re-examined the TMJ area at baseline (T0) and again at the six-month (T1) and twelve-month (T2) checkups after the utilization of the appliance.

## TMJ assessment procedure:

The protocol established by the Research Diagnostic Criteria for Temporomandibular Disorders (RDC/TMD) Axis I was used to check the function of the temporomandibular joint. It consisted of checking for clicking or creaking sounds in the joints, noticing tenderness over the joints and muscles, examining the range of motion of the jaw and asking patients about any pain or problems with how they use their jaw.

## Collecting data and statistical analysis:

A trained professional did all the clinical evaluations to ensure they were performed equally. The data were first stored in Microsoft Excel and processed using SPSS version 26.0 (IBM Corp., Armonk, NY, USA). Differences in TMJ parameters among the three time points were examined using a repeated-measures analysis of variance (ANOVA). Any p - value smaller than 0.05 was judged to be significant statistically.

## Results:

A total of 60 adolescents (30 males and 30 females) participated in the study and completed the 12-month follow-up. The mean age of the participants was 14.5 ± 1.7 years. The results were analyzed to observe changes in temporomandibular joint (TMJ) function across three assessment intervals: baseline (T0), 6 months (T1), and 12 months (T2). At the beginning of the study, 15 participants (25%) reported mild TMJ discomfort. By the 6-month evaluation, an increase in subjective symptoms was noted, with 20 participants (33.3%) experiencing discomfort. However, at the 12-month follow-up, only 6 subjects (10%) reported such symptoms. Joint sounds, particularly clicking, were noted in 10 participants (16.7%) at baseline, which rose to 18 (30%) at 6 months but decreased to 5 (8.3%) by the end of the study period. These findings suggest a temporary increase in TMJ symptoms during the active phase of orthodontic treatment, with improvement observed as treatment progressed (Table 1 - see PDF). A significant improvement in mandibular range of motion was observed across the study period. The mean maximum mouth opening increased from 38.4 ± 3.1 mm at T0 to 41.0 ± 2.9 mm at T2. Lateral excursions and protrusive movements also showed gradual improvement, which was statistically significant (p = 0.032) (Table 2 - see PDF). There were no cases of functional limitation at the final assessment. These findings indicate that while mild and transient TMJ symptoms may appear during the active phase of orthodontic treatment, there is a general trend toward resolution and improved mandibular function over time (Tables 1 and 2 - see PDF).

## Discussion:

There is no long-lasting damage to the temporomandibular joint (TMJ) from fixed orthodontic treatment in adolescents. There were brief but noticeable symptoms like increased joint noises and discomfort at the beginning of therapy which faded considerably by the 12th month. This finding goes along with evidence suggesting that braces or other orthodontic care do not worsen TMDs clinically [1, 2]. Orthodontically treated teens and those not treated have similar levels of TMD signs and symptoms, according to the findings of several studies [3, 4]. The short-lived symptoms noticed at the 6-month visit such as noises and gentle pain in the jaw, may be explained by neuromuscular changes resulting from the appliances [5, 6]. Most patients with these early signs improved without medical treatment by one year, suggesting that the changes were adaptive, not due to a disease [7]. Movement of the lower jaw and mouth increased as the treatment progressed. They may be caused by fixing occlusal problems and improving the way muscles and nerves function after an orthodontic treatment [8, 9]. The increased motion seen in normal movements of the mandible points out that having orthodontic treatment may help, not hinder, TMJ function in the years to come [10]. The association between orthodontic treatment and the development of temporomandibular disorders (TMD) remains a debated topic in the literature. Systematic reviews generally conclude that there is no definitive cause-effect relationship between orthodontic interventions and TMD onset. However, individual studies have indicated that extended use of fixed appliances, particularly during adolescence, may elevate the risk of developing TMD symptoms, suggesting a potential link under specific conditions [11]. Furthermore, the modality of orthodontic treatment may influence TMD outcomes. Evidence indicates that fixed appliance therapy is more frequently associated with increased TMD-related symptoms, whereas aligner-based treatments tend to have minimal or negligible effects on temporomandibular joint function [12]. These findings highlight the importance of individualized treatment planning and monitoring of temporomandibular health throughout orthodontic care. Because we used clinical tests and questioned the patients, we could get a clear picture of their TMJ health. Improvements in muscle sensation and joint sounds at the last check show the need for careful observation instead of jumping to conclusions about early symptoms [13]. Earlier papers raised questions about whether malocclusion treatment could lead to TMD, but recent studies and analyses have mostly disputed such ties [14, 15]. There is no proof that orthodontic treatment causes TMD from multiple studies. Other risk factors like psychological stress, using teeth in unusual ways and trauma are thought to play a bigger role in TMD than orthodontic treatment.

## Conclusion:

We show that a brace to correct teeth alignment in adolescence does not usually cause problems with the TMJ. Minor and self-limiting symptoms often happen during treatment and get better without lasting problems.
